# SHP2在非小细胞肺癌中的表达及意义

**DOI:** 10.3779/j.issn.1009-3419.2010.02.03

**Published:** 2010-02-20

**Authors:** 春兰 唐, 向东 周, 和平 杨, 清良 王, 容 章

**Affiliations:** 1 400038 重庆，第三军医大学西南医院呼吸内科 Department of Respiratory, Southwest Hospital, Third Military Medical University, Chongqing 400038, China; 2 400038 重庆，第三军医大学西南医院病理研究所 Department of Pathology, Southwest Hospital, Third Military Medical University, Chongqing 400038, China

**Keywords:** 肺肿瘤, 组织芯片, 免疫组化, SHP2, Lung neoplasms, Tissue microarray, Immunohistochemistry, SHP2

## Abstract

**背景与目的:**

以往研究表明, 异常的酪氨酸磷酸化与癌的发生密切相关, 本研究旨在采用组织芯片技术结合免疫组化方法来研究蛋白酪氨酸磷酸酶SHP2在非小细胞肺癌(non-small cell lung cancer, NSCLC)中的表达及意义。

**方法:**

80例NSCLC石蜡标本制成组织芯片, 采用链菌素亲生物素-过氧化物酶法(SP)进行免疫组化检测。

**结果:**

SHP2在NSCLC中的表达率为70.00%(56/80), 其中鳞癌为72.5%(29/40), 腺癌为67.50%(27/40);有无淋巴结转移的患者SHP2的阳性表达率分别为73.61%(53/72)和37.50%(3/8)(*P* < 0.05);SHP2的表达与患者性别、年龄、肿块大小、病理类型、分化程度、临床分期间无统计学差异(*P* < 0.05)。

**结论:**

SHP2在NSCLC中有较高的表达率, 且与淋巴结转移密切相关, 提示肺癌的发生、发展可能与SHP2有关, SHP2可能是肺癌新的标志物及治疗靶点。

## Abstract (en)

**Background and objective:**

Precious studies proven that aberrant tyrosine phosphorylation has linked with cancer.The aim of this work is to study the expression and significance of SHP2 in non-small cell lung cancer (NSCLC) through tissue microarray technique and immunohistochemical method.

**Methods:**

Eighty NSCLC specimens were constructed into tissue microarray and performed using immunohistochemistry.

**Results:**

The total positive rates of SHP2 were 70.7% (56/80) in NSCLC, 72.5% (29/40) in squamous cell carcinoma and 75% (27/40) in adenocarcinoma, which was not significant difference in sex, age, the size of tumor, histology, clinical stages and differentiation (*P* > 0.05), the positive rates of SHP2 were significantly higher in the cases with lymphnode metastasis than those without (*P* < 0.05).

**Conclusion:**

The expression rate of SHP2 is high and closely correlated to lymphnode metastasis in NSCLC, which implies the occurrence and development of lung cancer may be related to SHP2, and SHP2 may be a new marker and therapeutic targets for lung cancer.

蛋白酪氨酸磷酸化在细胞信号转导过程中具有重要作用, 酪氨酸磷酸化水平受控于蛋白酪氨酸激酶(protein tyrosine kinase, PTK)及蛋白酪氨酸磷酸酶(protein tyrosine phosphatase, PTP), 二者的失衡会导致异常的酪氨酸磷酸化, 而异常的酪氨酸磷酸化与人类多种疾病包括癌症的发生密切相关, 许多PTPs参与肿瘤的发生、发展, 因此是潜在的癌症药物治疗靶点^[1]^。SHP2(Src homology phosphotyrosyl phosphatase 2)为含有两个SH2(Src homology-2)域的蛋白酪氨酸磷酸酶, 是PTP超家族中第一个癌基因*SHP2*基因的蛋白产物, 在细胞生长、生存、侵袭、迁移、转化、形态发生中具有重要作用。研究^[2-5]^表明, SHP2的突变与努南综合征和豹斑综合征的发生密切相关, SHP2的活化突变也是多种血液系统肿瘤的病因, 且在乳腺癌中也有SHP2的高表达^[6]^, 但目前尚无SHP2与肺癌关系的研究报道。本文采用组织芯片及免疫组化技术, 以探讨SHP2在非小细胞肺癌(non-small cell lung cancer, NSCLC)中的表达情况, 现报道如下。

## 1 材料与方法

### 1.1 标本来源与处理

共收集2005年9月-2007年3月第三军医大学西南医院胸外科肺癌手术标本83例(其中鳞癌44例, 腺癌24例, 肺泡细胞癌8例, 腺鳞癌5例, 大细胞癌1例, 神经内分泌癌1例); 另从本院病理研究所档案材料中选取2003年-2004年手术肺癌石蜡包埋组织26例(其中腺癌21例, 鳞癌5例)。从上述109例病例中随机选取鳞癌40例, 腺癌40例, 另取正常肺组织10例。其中男性68例(鳞癌38例, 腺癌30例), 女性12例(鳞癌2例, 女性10例); 分化程度:Ⅰ级21例(其中鳞癌10例, 腺癌11例), Ⅱ级52例(其中鳞癌28例, 腺癌24例), Ⅲ级7例(其中鳞癌2例, 腺癌5例); 有淋巴结转移者72例(其中鳞癌37例, 腺癌35例), 无淋巴结转移者8例(其中鳞癌3例, 腺癌5例); 临床分期:Ⅰ期8例(其中鳞癌3例, 腺癌5例), Ⅱ期20例(其中鳞癌9例, 腺癌11例), Ⅲ期52例(其中鳞癌28例, 腺癌24例)。另选10例非肺部疾病死亡尸检肺组织作对照。患者术前均未经过化疗及放疗。

### 1.2 主要试剂

鼠抗人SHP2单克隆抗体购自Santa Cruz公司。组织芯片仪购自美国Beecher Instruments公司。载玻片(上海精轮工业玻璃有限公司)用1:10的多聚赖氨酸溶液处理。

### 1.3 方法

#### 1.3.1 组织芯片的制作

在HE切片上选择癌组织、癌旁组织各两点, 对照切片, 在石蜡块上进行相应标记。制作受体蜡块, 平整蜡块表面, 厚度以1.0 cm-1.2 cm为宜。把受体蜡块固定在固定槽中, 调节X、Y轴上微量标尺指示器为零, 调节组织芯片仪左边的距离定位螺丝, 确定打孔深度为3 mm, 然后向下打孔。移走细针, 用粗针对准预先做好的标记, 挖取供体组织。然后将粗针里的供体组织放入受体蜡块的孔中。全部点样完毕, 将组织芯片蜡块放入37 ℃烤箱中15 min。取出后, 用载玻片平整蜡块表面, 然后按照常规石蜡切片方法进行切片, 装盒备用。

#### 1.3.2 免疫组化染色及结果判定

采用SP法进行免疫组化染色。一抗为SHP2抗体。PBS替代一抗作空白对照, 正常肺组织作正常对照。按着色强度分为:阴性(-), 无阳性细胞; 弱阳性(+), 呈浅黄色; 中度阳性(++), 呈棕黄色; 强阳性(+++), 呈棕褐色。(+)-(+++)计为阳性。

### 1.4 统计学分析

采用SPSS 11.0统计软件包处理数据, 组间资料比较采用*χ*^2^检验, 以*P* < 0.05为差异有统计学意义。

## 2 结果

### 2.1 组织芯片构建情况

成功构建了含有340个点(每例各取癌组织2点, 癌旁组织2点, 正常肺组织每例各取2点)的NSCLC组织的微阵列蜡块, 由此切片得到NSCLC组织芯片(图 1)。各点组织的形态结构均有很好的代表性, 可反映原病理切片的结构特点。

**1:**
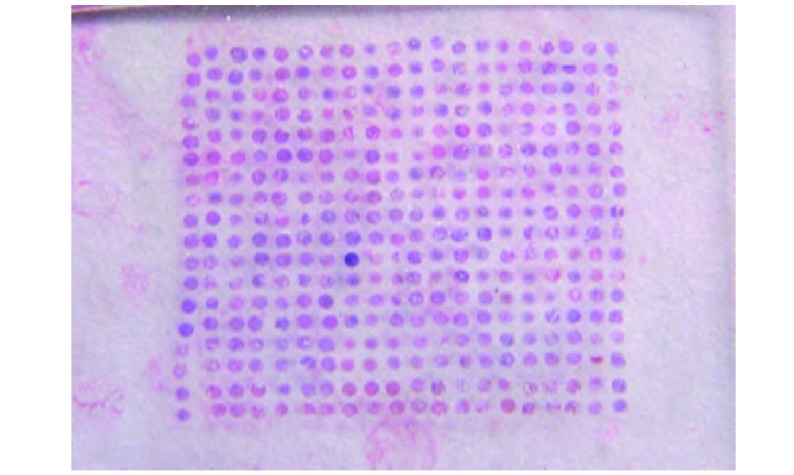
组织芯片 Tissue microarray

### 2.2 免疫组化结果

#### 2.2.1 SHP2染色结果

SHP2主要表达于肿瘤细胞的细胞质中(图 2), 癌旁组织和正常对照组无阳性表达。

**2:**
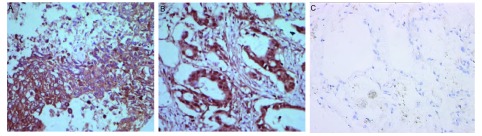
SHP2在鳞癌、腺癌和正常组织中的表达(SP×400) Expression of SHP2 in squamous cell carcinoma, adenocarcinoma and normal tissue (SP×400)

#### 2.2.2 SHP2阳性表达与性别、年龄、肿块大小、病理类型、分化程度、淋巴结转移、临床分期的关系

SHP2在男性/女性患者的阳性表达率分别为72.06%(49/68)和58.33%(7/12), 差异无统计学意义(*P*=0.495);在大于或等于/小于55岁的患者中, SHP2的阳性表达率分别为78.05%(32/41)和61.54%(24/39), 差异无统计学意义(*P*=0.144);在肿块大小上, 在肿块直径大于或等于/小于3 cm的患者中, SHP2的阳性表达率分别为70.66%(53/75)和60.00%(3/5), 差异无统计学意义(*P*=0.633);SHP2在鳞癌/腺癌的阳性表达率为分别为72.50%(29/40)和67.50%(27/40), 差异无统计学意义(*P*=0.808);分化程度为Ⅰ+Ⅱ级/Ⅲ级的患者SHP2的阳性表达率分别为69.86%(51/73)和71.43%(5/7), 差异无统计学意义(*P*=1.000);临床分期为Ⅰ+Ⅱ期/Ⅲ期的患者SHP2的阳性表达率分别为57.14%(16/28)和76.92%(40/52), 差异无统计学意义(*P*=0.078);有/无淋巴结转移的患者SHP2的阳性表达率分别为73.61%(53/72)和37.50%(3/8), 有统计学差异(*P*=0.048)。提示MMP-2、MMP-9阳性表达与NSCLC患者的性别、年龄、肿块大小、病理类型、分化程度、临床分期无关, 而与淋巴结转移相关(见表 1)。

**1:** SHP2的表达水平与非小细胞肺癌临床病理特征的关系 The relationship between SHP2 expression and clinicopathological characteristics in non-small cell lung cancer

Characteristics	*n*	SHP2		
		Positive cases	Positive rate (%)	*P*
Sex				0.495
Male	68	49	72.06	
Female	12	7	58.33	
Age (year)				0.144
> 55	41	32	78.05	
< 55	39	24	61.54	
The size of tumor				0.633
> 3 cm	75	53	70.66	
< 3 cm	5	3	60.00	
Histology				0.808
Squamous cell carcinoma	40	29	72.50	
Adenocarcinoma	40	27	67.50	
Differentiation				1.000
Ⅰ+Ⅱ	73	51	69.86	
Ⅲ	7	5	71.43	
Lymphnode metastasis				0.048
Yes	72	53	73.61	
No	8	3	37.50	
Stage				0.078
Ⅰ+Ⅱ	28	16	57.14	
Ⅲ	52	40	76.92	

## 3 讨论

肺癌是全球发病率和死亡率最高的恶性肿瘤, 其中约有80%为NSCLC。尽管近年来对肺癌的病因、发病机制、诊断及治疗取得了一定进展, 但目前肺癌的5年生存率却仍然较低, 因此, 探索肺癌的病因、发病机制、寻找新的治疗靶点及标志物具有重要意义。

研究^[3, 4]^表明, SHP2的活化突变是青少年粒-单核细胞性白血病、髓细胞性白血病、慢性粒-单核细胞性白血病的病因; 它也参与幽门螺旋杆菌相关胃癌的发生^[7]^; 在乳腺癌中也有SHP2的高表达(72%), 且SHP2的表达与乳腺癌的淋巴结转移、分化程度密切相关, 有淋巴结转移、分化程度越低的患者SHP2的表达越高, 在SHP2过表达的乳腺癌病例, HER2也过表达^[6]^, 且抑制SHP2的表达可使间充质细胞向正常上皮细胞转化^[8]^。因此, SHP2有望成为多种白血病及实体瘤包括乳腺癌新的标志物及治疗靶点, 目前也已开展SHP2抑制剂的研究^[9]^, 但至今尚无SHP2与肺癌关系的研究报道。

组织芯片技术最早由Kononen等于1998年建立并报道, 具有高产出、实验误差小、方便经济、对原始组织蜡块损坏小等优点, 现已得到广泛的应用, 研究内容包括膀胱移行细胞癌、结直肠癌、肺癌、人脑胶质细胞瘤。本实验共选取80例NSCLC患者的肺癌标本制作组织芯片, 结合免疫组化方法, 首次方便、快速、高效地检测了SHP2在NSCLC中的表达, 并探讨其与肺癌患者性别、年龄、肿块大小、组织类型、分化程度和有无淋巴结转移以及临床分期的关系。

本研究结果显示, SHP2在NSCLC中有较高阳性表达率[70.00%(56/80)], 其中鳞癌为72.5%(29/40), 腺癌为67.50%(27/40), 而在癌旁组织及正常肺组织中无表达, 提示SHP2在NSCLC中的表达具有较高的特异性和敏感性, SHP2可作为NSCLC新的标志物, 且可能与预后相关。进一步分析发现, SHP2在NSCLC中的表达与性别、年龄、肿块大小、组织类型无关, 提示未来针对SHP2靶点的NSCLC治疗有较广泛的适宜人群。

肺癌患者的高死亡率与远处脏器转移如肝、骨、脑、心包转移密切相关, 而淋巴结状态是预测转移的有效手段之一。本研究结果显示, SHP2在有淋巴结转移组的阳性表达率显著高于无淋巴结转移组, 尽管目前没有SHP2促进肿瘤转移的在体数据, 但许多体外研究^[10-13]^表明, SHP2可促进细胞的运动、粘附及迁移, SHP2在细胞生长、生存、转化、形态发生中的也具有重要作用, 提示SHP2可能与肺癌的发生、发展密切相关。结合SHP2在NSCLC中的特异高表达, 进一步提示SHP2有可能成为肺癌新的治疗靶点及预后标志物, 进一步研究其与肺癌的关系有望成为肺癌研究领域新的热点。值得注意的是, 本研究发现SHP2的表达虽与淋巴结转移相关, 但却与临床分期及分化程度无关, 这似乎矛盾, 但从表达率来看, Ⅲ期NSCLC仍明显高于Ⅰ+Ⅱ期NSCLC, 统计学无差异可能与病例数不够多和病例分期及病理分级不平衡有关, 今后可扩大病例数进一步研究。
